# SEM, confocal Laser, and histological evaluation of traditional and conservative access cavities with and without 3D cleaning: an ex vivo study

**DOI:** 10.1007/s00784-025-06606-9

**Published:** 2025-10-11

**Authors:** Dina Abdellatif, Alfredo Iandolo, Spagnuolo Gianrico, Carlo Rengo, Mariangela Cernera, Christophe Meyer, Davide Mancino

**Affiliations:** 1 Department of Oral Rehabilitation, Faculty of Dental Surgery, University of Marie and Louis Pasteur, Besançon, France; 2 Department of Nanomedicine, Imaging, Therapeutics, Laboratory Sinergies (UR 4662), University of Marie and Louis Pasteur, Besançon, France; 3 Department of Maxillo Facial Surgery and Dentistry, CHU, CESD, Hospital and University Centre Minjoz, Besançon, France; 4 Department of Neurosciences, Reproductive and Odontostomatological Sciences, University of Naples “Federico II”, Napoli, Italy; 5 Department of Nanomedicine, Imaging, Therapeutics, Laboratory Sinergies, University of Marie and Louis Pasteur, Besançon, France; 6 Department of Maxillo Facial Surgery and Dentistry, CHU, Hospital and University Centre Minjoz, Besançon, France; 7 Department of Biomaterials and Bioengineering, INSERM UMR_S 1121, Strasbourg University, Strasbourg, France; 8 Department of Endodontics, Faculty of Dental Medicine, Strasbourg University, Strasbourg, France; 9 Department of Maxillo Facial Surgery and Dentistry, University Hospital, Strasbourg, France

**Keywords:** Access cavity, Cleaning, Endodontics, NaOCl, Ultrasonics

## Abstract

**Objective:**

The present investigation sought to compare the efficacy of a 3D Cleaning protocol comprising intrachamber sodium hypochlorite (NaOCl) preheating followed by ultrasonic activation on smear layer and debris removal, residual debris, and NaOCl penetration under different access cavity designs (traditional versus conservative). To the best of our knowledge, this is the first study to evaluate irrigant activation across access cavity designs within a single, standardised experimental framework.

**Materials and methods:**

Specimens were allocated to three primary groups according to the analytical method: Group A, scanning electron microscopy (SEM); Group B, histological analysis; and Group C, confocal laser scanning microscopy (CLSM). Each group was further subdivided into four subgroups (*n* = 10 per subgroup): A1/B1/C1 = traditional access without 3D Cleaning; A2/B2/C2 = traditional access with 3D Cleaning; A3/B3/C3 = conservative access without 3D Cleaning; A4/B4/C4 = conservative access with 3D Cleaning. Smear layer/debris removal, residual debris, and NaOCl penetration were assessed via SEM, histology, and CLSM, respectively. Data were analysed using the Kruskal–Wallis H test with Dunn-adjusted post hoc comparisons (α = 0.05).

**Results:**

Subgroups subjected to 3D Cleaning (A2, A4; B2, B4; C2, C4) demonstrated significantly lower smear layer/debris scores and greater NaOCl penetration compared with their respective controls (A1, A3; B1, B3; C1, C3) (*p* < 0.05). Histological assessment (Group B) revealed reduced residual debris in 3D Cleaning subgroups (B2, B4) relative to B1 and B3. CLSM analysis (Group C) confirmed markedly deeper irrigant penetration in C2 and C4 versus C1 and C3. With respect to access design, 3D Cleaning enhanced outcomes irrespective of approach; conservative access combined with 3D Cleaning frequently produced the most favourable results, although differences compared with traditional access plus 3D Cleaning were modest and not consistently significant (e.g., C4 vs. C2: +17 μm; 95% CI − 2.31 to + 36.31).

**Conclusions:**

A single, standardised 3D Cleaning protocol significantly improved debridement and irrigant penetration regardless of access cavity design. Conservative access combined with 3D Cleaning frequently yielded the most advantageous outcomes.

**Clinical relevance:**

Given that the protocol relies on commonly available equipment and straightforward procedures, 3D Cleaning represents a pragmatic adjunct to overcome potential cleaning limitations associated with conservative access. Nevertheless, the findings are derived from ex vivo experimentation and require validation in clinical settings.

**Supplementary Information:**

The online version contains supplementary material available at 10.1007/s00784-025-06606-9.

## Introduction

Endodontic therapy encompasses diagnosis, access preparation, canal shaping, chemical cleaning, and obturation, with chemical cleaning regarded as pivotal for the debridement of complex anatomies and dentinal tubules [1]. Since microorganisms and necrotic tissue frequently persist in areas inaccessible to mechanical instruments, active irrigation is essential for achieving effective disinfection.

Mechanical instrumentation of root canals inevitably generates a smear layer, typically 2–5 μm thick, composed of organic and inorganic components such as dentine particles, pulp remnants, bacteria, and microbial by-products [2, 3]. In infected canals, this smear layer serves as a reservoir for pathogens within dentinal tubules, impeding the penetration of irrigants and intracanal medicaments, while also compromising the sealing ability of obturation materials. These effects increase the risk of coronal and apical microleakage [3, 4]. Consequently, removal of the smear layer is critical to optimise disinfection, including within lateral canals and dentinal tubules, thereby significantly enhancing treatment outcomes [5].

Conventional irrigation protocols employ sodium hypochlorite (NaOCl) and ethylenediaminetetraacetic acid (EDTA) [6, 7]. However, the efficacy of these regimens depends strongly on the mode of irrigant delivery and activation [8, 9]. Ultrasonic and sonic agitation, negative-pressure systems, and laser-assisted irrigation have all been proposed, yet each approach has potential limitations. These range from operator-dependent tip positioning and the risk of apical extrusion under positive pressure to the financial and training demands associated with laser systems. Such considerations highlight the clinical need for simple and widely adoptable activation strategies.

Concurrently, the paradigm of minimally invasive endodontics advocates conservative access cavity designs to preserve coronal structure and improve fracture resistance. However, conservative access may restrict irrigant hydrodynamics and thereby increase residual debris and smear layer, unless offset by effective activation. The 3D Cleaning protocol—comprising intrachamber warming of NaOCl to approximately 80 °C immediately followed by ultrasonic activation—has emerged as a streamlined technique using standard clinical equipment, potentially counterbalancing hydrodynamic limitations while preserving tooth structure [10–12].

NaOCl heating and ultrasonic activation are both supported by the literature. However, conservative access may reduce cleaning efficacy, and no previous study has directly compared irrigant activation outcomes across different access cavity designs within a unified, standardised experimental framework. Accordingly, the modifying effect of access geometry on activation performance remains unclear.

The aim of this study was therefore to compare smear layer and debris removal, as well as irrigant penetration, between traditional and conservative access cavities with and without 3D Cleaning. The null hypothesis tested was that no significant differences would exist among groups with respect to these cleaning parameters.

##  Materials and methods

The study was conducted in accordance with the principles of the Declaration of Helsinki and was approved by the Institutional Review Board of the University of Strasbourg, France (protocol code CE-2025-16).

### Sample size and specimens

#### **Sample size calculation**

A priori sample size estimation was performed using G*Power software, targeting the primary outcome (ordinal smear layer score). In the absence of directly comparable variance data, a medium effect size (Cohen’s *f* = 0.40) was assumed, consistent with multi-group ex vivo studies of activated versus non-activated irrigation. With α = 0.05, power = 0.80, and *k* = 4 subgroups per analytical modality, the minimum requirement was *n* = 40 per modality (SEM, histology, CLSM), i.e., 10 teeth per subgroup, giving a total *N* = 120. This calculation was considered conservative to accommodate potential variance inflation across access cavity designs.

#### **Specimen selection**

One hundred and twenty extracted human mandibular molars were included. These teeth were selected for their anatomical relevance to access cavity design and their prevalence in endodontic practice. All specimens had been extracted for periodontal reasons unrelated to this study.

### Inclusion and exclusion criteria

Extracted human mandibular molars with fully formed apices and intact crowns were included. Teeth were excluded if they exhibited cracks or fractures, root resorption, previous endodontic or restorative treatment, calcifications, or open apices. Anatomical screening was performed using standardised orthogonal periapical radiographs (mesio-distal and bucco-lingual projections) and canal scouting with #10 K-files. Canal curvature was quantified on the mesio-distal projection using the Schneider method; only teeth with moderate curvature (approximately 10°–30°) were accepted.

In this ex vivo context, cone-beam computed tomography (CBCT) was not employed; two-plane radiography combined with canal scouting was deemed sufficient to operationalise “normal anatomy,” defined as fully formed apices, absence of resorption or calcification, and canal configurations typical of mandibular molars. Following extraction, periodontal tissues were mechanically removed from external surfaces, and specimens were stored in physiological saline at 4 °C until experimentation.

### Root canal preparation and irrigation

Root canals were instrumented with HyFlex EDM files (Coltene/Whaledent Inc., Cuyahoga Falls, OH, USA) in the sequence 10/0.05 → 20/0.05 → 30/0.04 to full working length. Apical patency was maintained using a #10 K-file (Coltene/Whaledent Inc.).

Irrigation during shaping was performed with 5 mL of 5.25% sodium hypochlorite (NaOCl; CanalPro™, Coltene/Whaledent, Altstätten, Switzerland) delivered through a 30-G side-vented needle (Coltene/Whaledent Inc.), followed by 2 mL of physiological saline (0.9% NaCl; S.A.L.F. S.p.A.—Laboratorio Farmacologico, Bergamo, Italy). A final rinse was performed with 3 mL of 17% EDTA (CanalPro, Coltene/Whaledent Inc.) for 1 min, followed by 2 mL of saline.

For CLSM analysis, NaOCl (CanalPro™ 5.25%) was supplemented with 0.1% Rhodamine B (analytical grade, CAS 81–88-9; Sigma-Aldrich, Merck KGaA, Darmstadt, Germany). Heat activation was performed with System-B (Analytic Endodontics, Orange, CA, USA), and ultrasonic activation employed Ultra Smart AI with a size 25.00 tip (COXO, Foshan, China). Histological and CLSM examinations were conducted using an Optika microscope (Turin, Italy).

### Operator and bias mitigation

A single experienced endodontist undertook all procedures (access, shaping, irrigation, and activation) to minimise inter-operator variability. Outcome assessors were blinded to group allocation. The analysis plan and effect-size reporting strategy were pre-specified.

### Randomisation and allocation concealment

Within each analytical modality (SEM, histology, CLSM), specimens were randomly assigned (1:1:1:1) to four experimental subgroups using Random Allocation Software 2.0, with permuted blocks and stratification by modality. The allocation sequence was generated by an independent researcher not involved in specimen preparation or treatment. Concealment was maintained with opaque, sequentially numbered envelopes, opened immediately prior to access preparation.

### Experimental groups

Specimens were allocated to one of three analytical modalities:

- **Group A** – Scanning electron microscopy (SEM).
- **Group B** – Histological examination.
- **Group C** – Confocal laser scanning microscopy (CLSM).

Each group was further subdivided into four experimental subgroups (*n* = 10 per subgroup):

- A1/B1/C1 – Traditional access without 3D Cleaning.
- A2/B2/C2 – Traditional access with 3D Cleaning.
- A3/B3/C3 – Conservative access without 3D Cleaning.
- A4/B4/C4 – Conservative access with 3D Cleaning.

Using Random Allocation Software 2.0, ensuring allocation concealment:

- Subgroups A1, B1, C1: Traditional access with conventional irrigation.
- Subgroups A2, B2, C2: Traditional access with 3D cleaning.
- Subgroups A3, B3, C3: Minimally invasive access with conventional irrigation.
- Subgroups A4, B4, C4: Minimally invasive access with 3D cleaning.

### Access cavity preparation

**Fig. 1:**
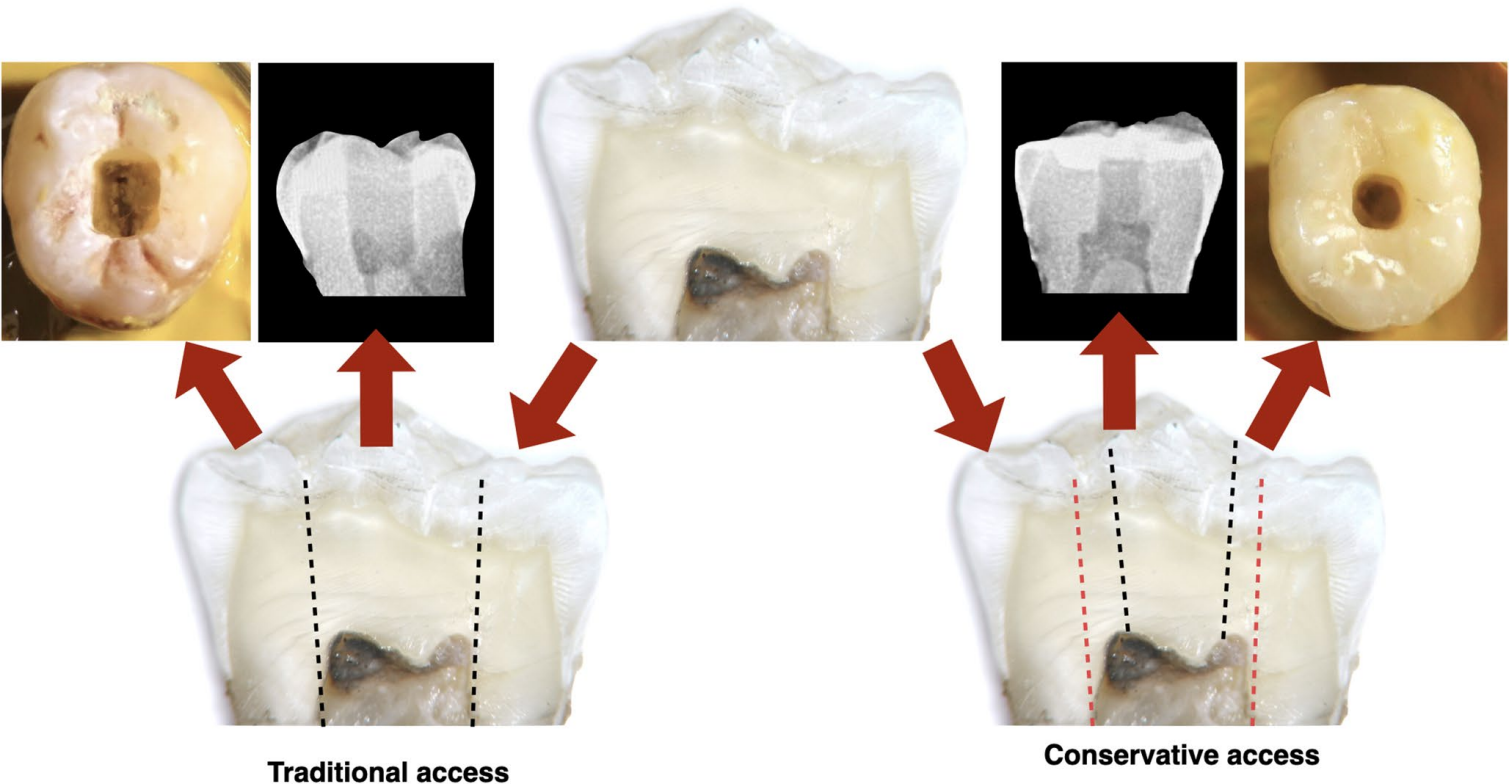
In the schematic illustration, the traditional access cavity involves complete removal of the chamber roof, whereas the conservative access cavity preserves portions of the roof, thereby maintaining greater dentine structure

**Fig. 2:**
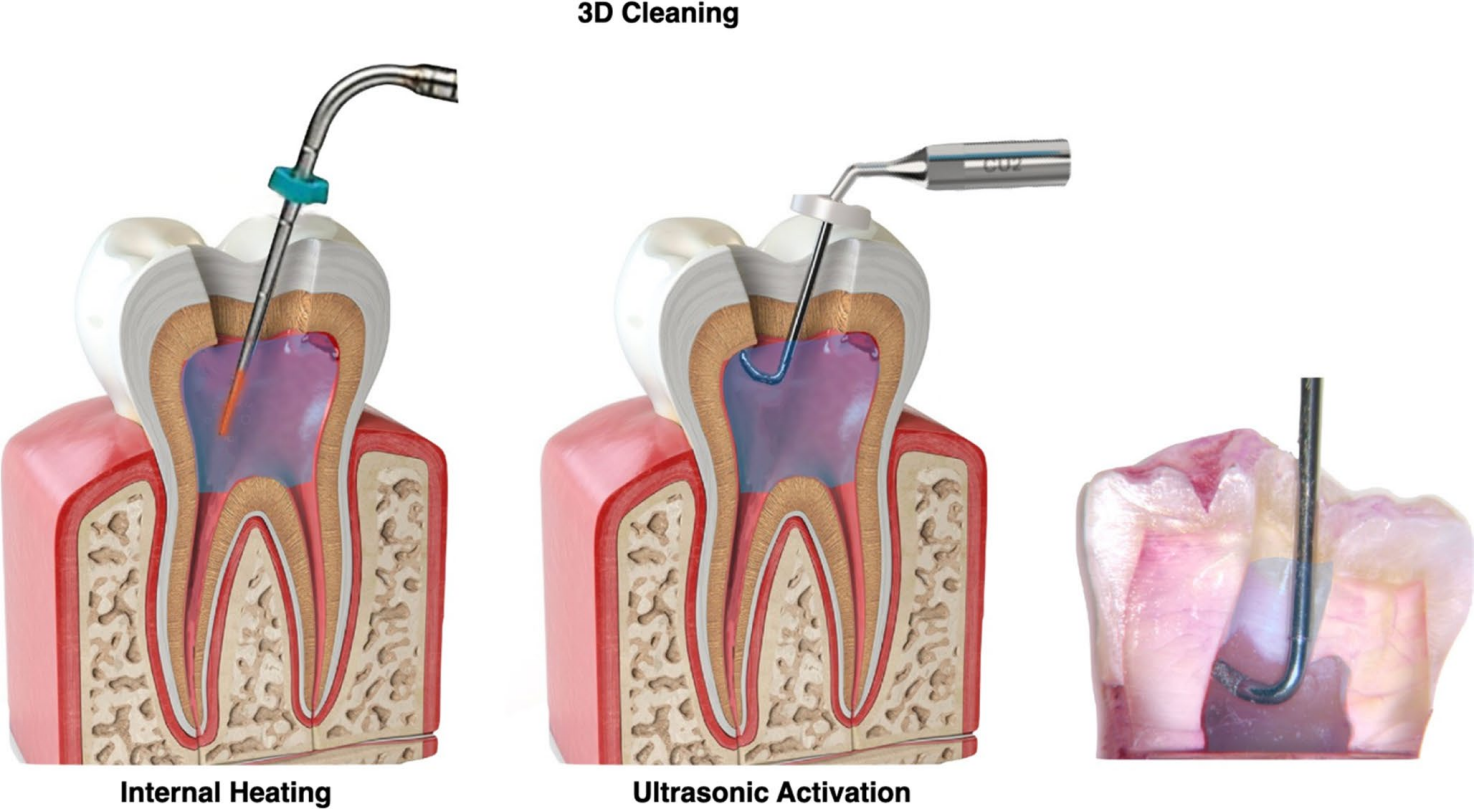
A schematic image of 3D cleaning protocol

- **Traditional access group.** Access outlines were extended to remove the entire chamber roof and to provide straight-line entry to all canal orifices. Preparations were refined with a round diamond bur (0.16 mm diameter, FG, high-speed; Komet Dental, Gebr. Brasseler GmbH & Co. KG, Lemgo, Germany) and a non-end-cutting bur (Endo-Z, FG, high-speed; Dentsply Maillefer, Ballaigues, Switzerland) to eliminate undercuts. Following preparation, canals were shaped and irrigated using the standard protocol and dried with sterile paper points (Coltene/Whaledent Inc., Cuyahoga Falls, OH, USA) (Fig. 1).
- **Traditional access + 3D Cleaning group.** Access cavity and root canals preparations were performed as above, followed by intra-chamber heating of NaOCl and ultrasonic activation (3D Cleaning protocol). NaOCl was introduced to the level of the occlusal surface. A System-B Heat Source (Analytic Endodontics, Orange, CA, USA) set at 180 °C with an X-fine tip (30/04) positioned 1 mm above the chamber floor (without dentine contact) was activated for 8 s, immediately followed by 20 s of ultrasonic activation with an Ultra Smart AI device (COXO, Foshan, China; size 25.00 tip). The cycle was repeated three times with fresh NaOCl for each iteration, then flushed with 2 mL distilled water and dried with paper points (Fig. 2).
- **Minimally invasive access group.** An orifice-directed approach was employed to conserve peri-cervical dentine and retain as much chamber roof as possible. The outline was restricted to triangulation lines between canal orifices, with extensions only as required for reproducible glide-path verification. Preparation was performed under magnification (OMS 3200, Zumax Medical Co., Ltd., Suzhou, China) and coaxial illumination. Canal orifices were confirmed with a DG-17 explorer (Hu-Friedy Group, Chicago, IL, USA) and #10 K-files. Root canal shaping and irrigation followed the same protocol as for the traditional group (Fig. 1).
- **Minimally invasive access + 3D Cleaning group.** This group combined conservative cavity preparation with the 3D Cleaning protocol described above (Fig. 2).

### SEM evaluation

**Fig. 3:**
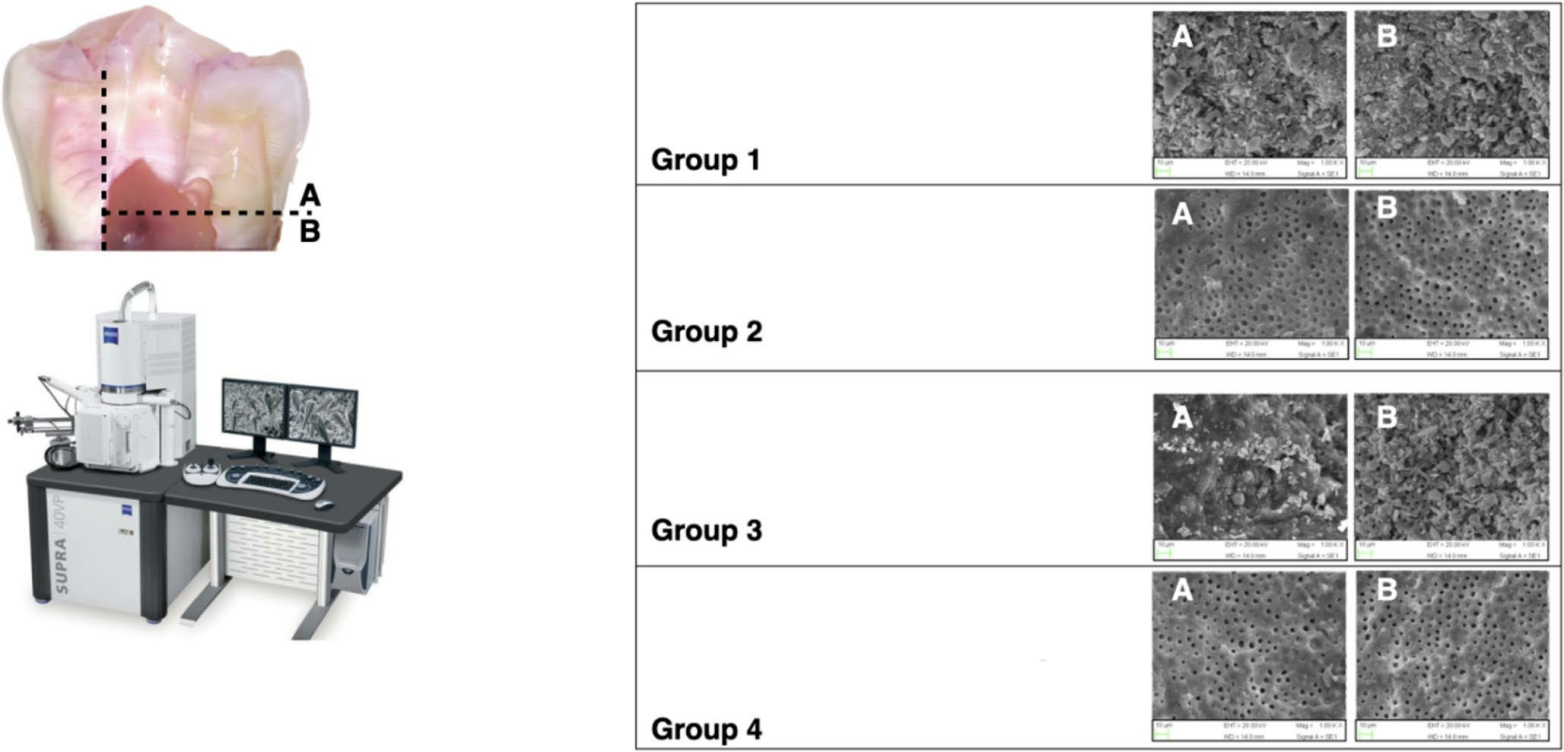
Representative scanning electron microscopy (SEM) images of the pulp chamber after access cavity preparation and cleaning protocols (×1000 magnification). **Group 1**: Traditional access cavity with conventional cleaning. **Group 2**: Traditional access cavity with 3D Cleaning. **Group 3**: Conservative access cavity with conventional cleaning. **Group 4**: Conservative access cavity with 3D cleaning

After treatment, each specimen was sectioned coronally at the cemento-enamel junction using a water-cooled diamond wafering blade. Two shallow mesiodistal longitudinal grooves were then created on the crown using a high-speed, water-cooled diamond bur to guide fracture and separate the crown halves, thereby exposing the pulp chamber and chamber walls. Specimens were submerged in liquid nitrogen and fractured along the grooves with a stainless-steel chisel, then air-dried and gold sputter-coated. SEM imaging was performed at ×1000 magnification; 20 micrographs per specimen (10 from coronal chamber-adjacent areas, 10 from apical regions) were acquired (Fig. 3).

#### **Scoring**

Three blinded evaluators graded smear layer and debris removal using Hulsmann’s criteria [13]:

- *Debris*: 1 = clean, 5 = walls fully covered.
- *Smear layer*: 1 = tubules open, 5 = heavy smear layer.

### Histological evaluation

Specimens were fixed in 4% buffered formalin for 48 h, rinsed, and decalcified in Morse’s solution for 28 days (refreshed every 48 h). Serial sections (~ 6 μm) from the access cavity region were stained with haematoxylin–eosin and examined using an optical microscope (OptiKa TB 290, Optika, Turin, Italy) at ×40, ×100, and ×200 magnification, with Optika Vision Lite software.

#### **Scoring**

Two blinded examiners graded residual debris:

- Grade 1 = debris throughout;
- Grade 2 = > 50% area covered;
- Grade 3 = > 25% area covered;
- Grade 4 = debris absent or < 25% area.
- A third examiner resolved disagreements through consensus.

### CLSM evaluation

External root surfaces were coated with nail varnish to prevent irrigant leakage. NaOCl (5.25%, CanalPro™, Coltene/Whaledent, Altstätten, Switzerland) was labelled with 0.1% Rhodamine B. After irrigation, cavities were dried with paper points, and specimens were sectioned into ~ 0.5 mm slices corresponding to SEM/histology levels. Sections were polished to ~ 40 μm thickness with wet silicon carbide papers.

**Fig. 4:**
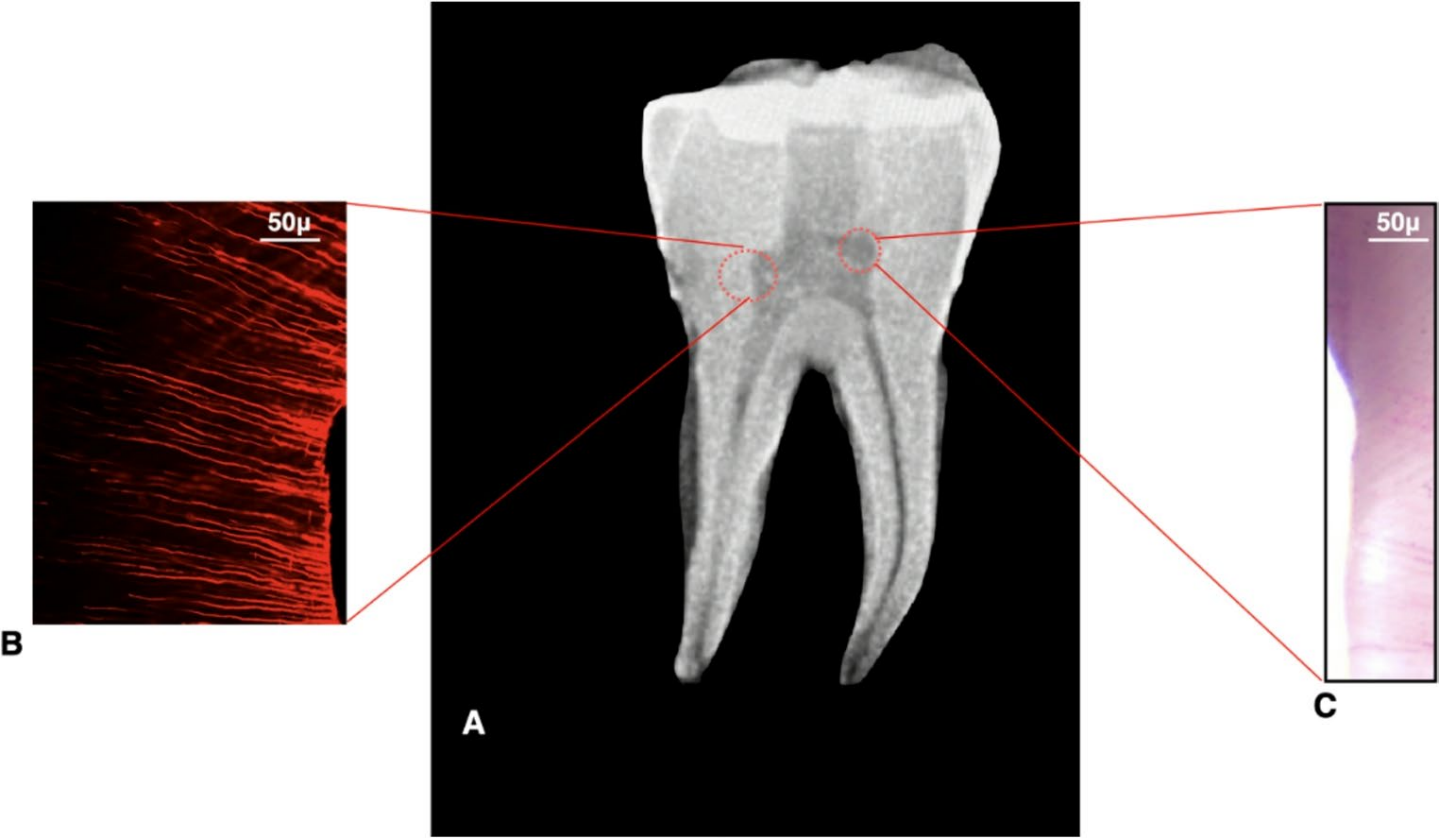
Representative images from histological and confocal laser scanning microscopy (CLSM) analyses. (**A**) Mandibular molar with a minimally invasive access cavity, demonstrating preservation of the pulp chamber roof. (**B**) CLSM image obtained following the 3D Cleaning protocol, illustrating irrigant penetration. (**C**) Histological section after application of the 3D Cleaning protocol, showing reduced residual debris

Specimens were examined under CLSM (LSM 800 with Airyscan, Carl Zeiss Microscopy GmbH, Jena, Germany) at ×40 and ×100 magnification, excitation 540–570 nm. The depth of NaOCl penetration into dentinal tubules was measured using ZEN software (Blue edition; Carl Zeiss Microscopy GmbH, Jena, Germany) (Fig. 4).

### Blinding, calibration, and inter-rater reliability

#### **Calibration and scoring**

Prior to formal evaluation, all examiners underwent structured calibration sessions using a reference set of SEM micrographs, histological sections, and CLSM image stacks spanning the full range of scoring criteria. Anchor images and operational definitions were agreed upon during a consensus meeting, followed by pilot scoring on a training set to stabilise application of the criteria.

SEM and histology ratings were subsequently performed independently and blinded to both group allocation and irrigation protocol. All images were anonymised and randomised before scoring. A third examiner adjudicated disagreements to achieve consensus. For CLSM, penetration-depth measurements were obtained by a single calibrated examiner blinded to group allocation; repeatability was verified by re-measuring a random subset.

#### **Reliability analysis**

Inter-rater agreement for SEM and histology (ordinal variables) was quantified with Cohen’s weighted κ (quadratic weights, 95% CI). For CLSM penetration depth (continuous variable), reliability was assessed using a two-way random-effects intraclass correlation coefficient, ICC(2,1), with 95% CI. Intra-rater reliability was established by re-scoring a random subset after a ≥ 2-week washout period, using the same metrics. The agreement was interpreted according to standard qualitative categories (fair, moderate, substantial, and almost perfect), with a prespecified acceptability threshold of substantial or higher.

All reliability analyses were performed after finalisation of the scoring rubric, while examiners and the data analyst remained blinded to allocation. Agreement statistics (weighted κ and ICC(2,1) with 95% CI) are reported in the Results and Supplementary material; all exceeded the prespecified “substantial” threshold.

### Statistical analysis

Data distributions were assessed for normality (Shapiro–Wilk test) and homogeneity of variances (Levene/Brown–Forsythe tests). Given violations of parametric assumptions, between-group differences were analysed with the Kruskal–Wallis H test, followed by Dunn’s post hoc comparisons with adjusted p-values (α = 0.05).

Effect sizes were reported as epsilon-squared (ε²) for omnibus tests and Hedges’ *g* (95% CI) for pairwise contrasts. Non-parametric confidence intervals were generated using bias-corrected and accelerated bootstrapping where applicable.

## Results

### **Overall comparisons**

Significant between-group differences were observed for all outcomes: SEM debris score, SEM smear-layer score, histological residual debris (%), and CLSM NaOCl penetration depth (µm) (Kruskal–Wallis H tests, all *p* < 0.05; full results in Tables 1, 2 and 3).

### SEM (debris and smear layer)

**Fig. 5:**
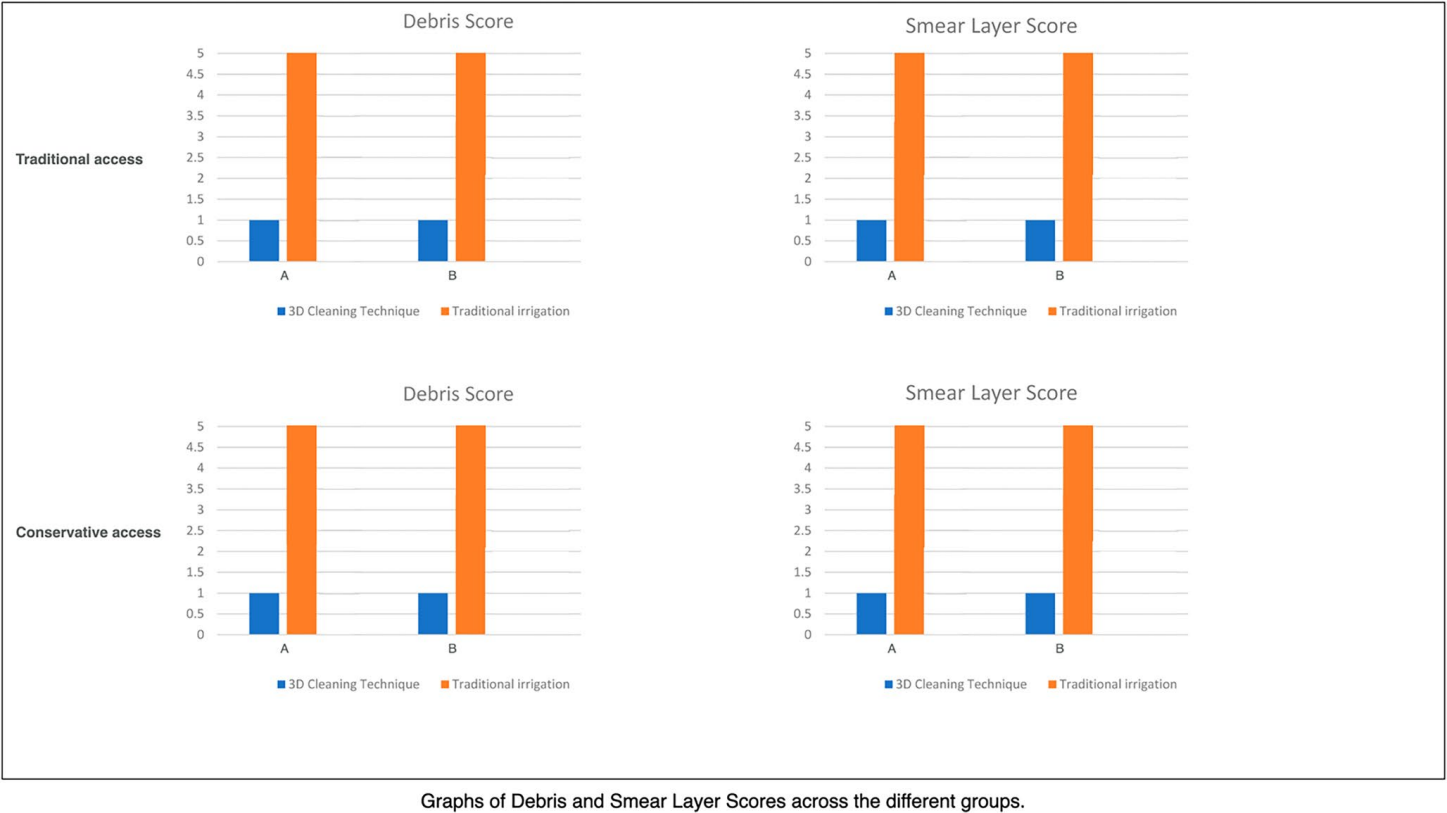
Graphs of debris and smear layer scores across the different groups

 Across both access designs, subgroups treated with 3D Cleaning showed significantly lower debris and smear-layer scores compared with non-activated controls (Dunn-adjusted *p* < 0.05). The conservative access + 3D Cleaning subgroup (A4) demonstrated the lowest overall scores (Table 1; Figs. 3 and 5).

### Histology (residual debris)

Residual debris was markedly reduced with 3D Cleaning:

- B2 = 12.4 ± 3.1% vs. B1 = 45.8 ± 6.2% (*p* < 0.05),
- B4 = 10.8 ± 2.9% vs. B3 = 47.9 ± 5.8% (*p* < 0.05).

**Fig. 6:**
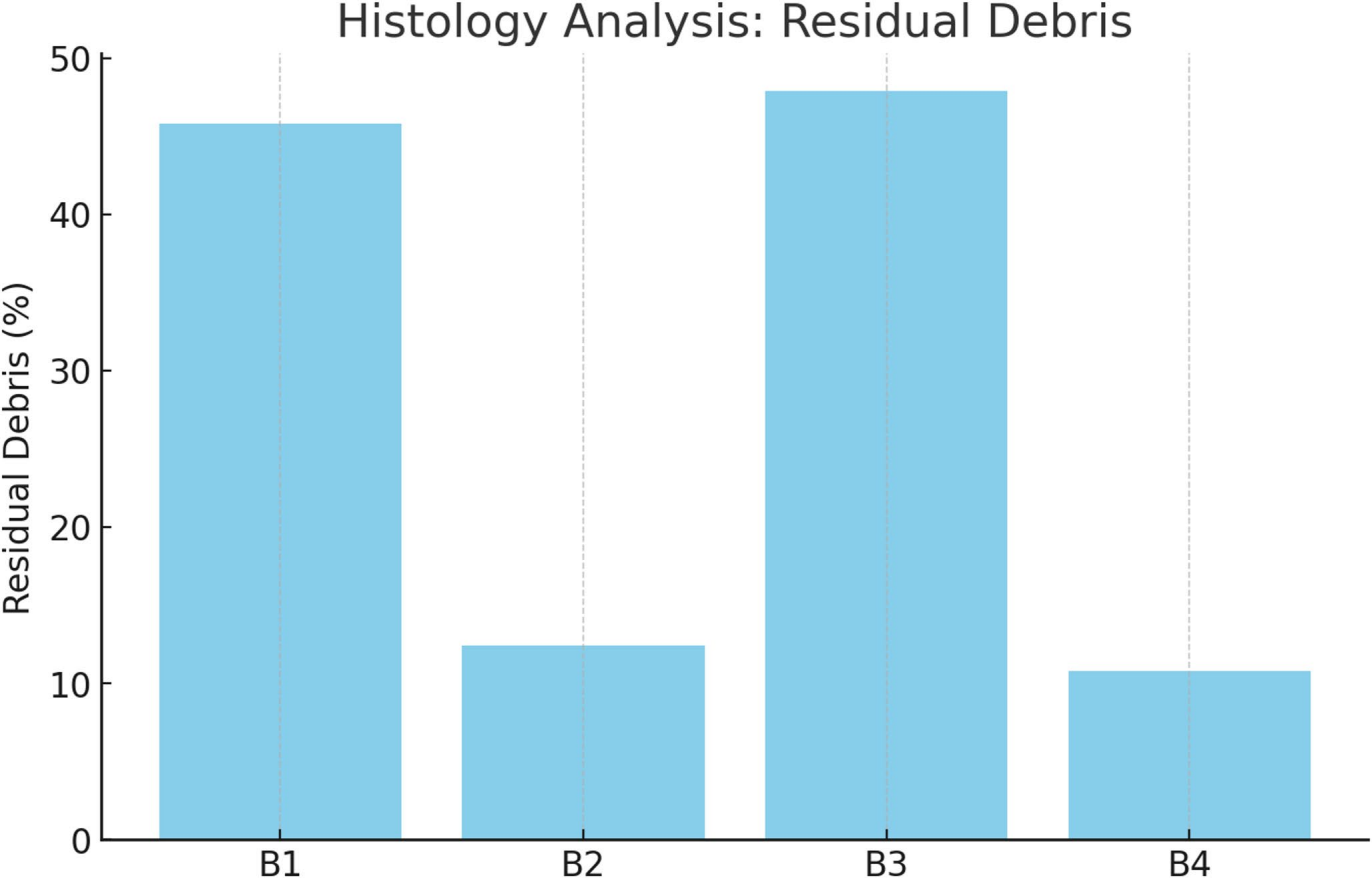
Histogram illustrating the percentage area of residual debris observed in histological sections. The 3D cleaning groups (B2, B4) exhibited markedly reduced residual debris compared to their respective controls (B1, B3), highlighting enhanced cleaning efficiency

Pairwise mean differences (Δ) and Hedges’ *g* effect sizes (95% CI) are detailed in Table 2 and Supplementary Table S1 (e.g., B2–B1 Δ = −33.40% [− 38.13, − 28.67]; B4–B3 Δ = −37.10% [− 41.53, − 32.67]) (Fig. 6).

### CLSM (NaOCl penetration)

Irrigant penetration depth was substantially greater in the 3D Cleaning subgroups:

- C2 = 258 ± 22 μm vs. C1 = 75 ± 15 μm,
- C4 = 275 ± 19 μm vs. C3 = 70 ± 12 μm (*p* < 0.05).

**Fig. 7:**
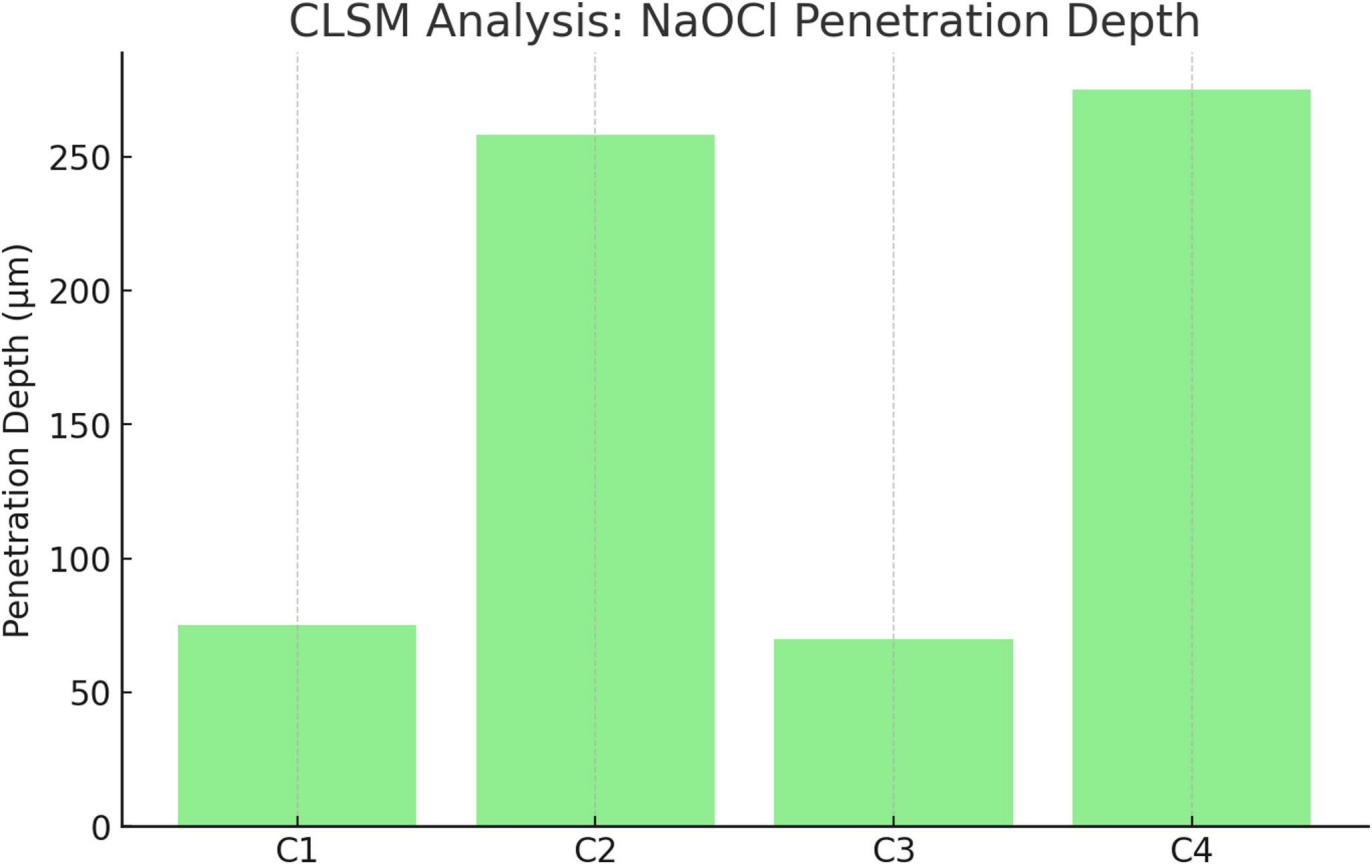
Sodium hypochlorite (NaOCl) penetration depth into dentinal tubules assessed by confocal laser scanning microscopy (CLSM). Groups treated with 3D Cleaning (C2, C4) exhibited significantly greater penetration depths, confirming enhanced irrigant diffusion, whereas conventional irrigation groups (C1, C3) demonstrated limited penetration

Conservative access + 3D Cleaning (C4) showed slightly deeper penetration than traditional access + 3D Cleaning (C2), although the difference was small and not consistently significant (C4–C2 Δ = +17 μm; 95% CI − 2.31 to + 36.31) (Table 3; Fig. 7, Supplementary Table S1).

**Table 1:**
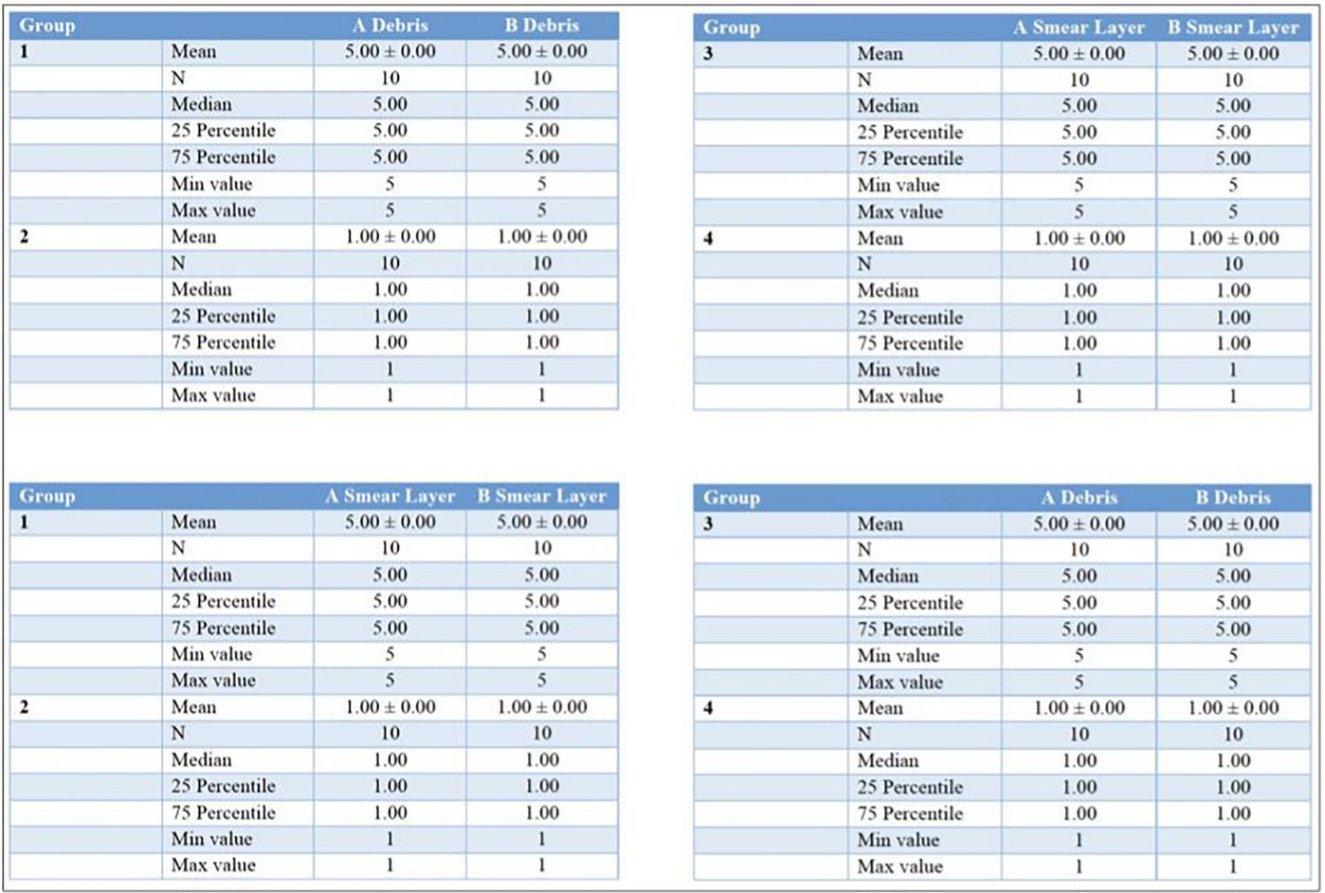
Means, medians, minimum and maximum scores per group

Central tendency and dispersion of SEM debris and smear-layer scores by experimental group. Values are presented as means, medians, minima, and maxima. Omnibus between-group differences were analysed using the Kruskal–Wallis H test; effect size is expressed as epsilon-squared (ε²) with 95% confidence intervals. Detailed pairwise comparisons (Dunn’s post hoc with adjusted *p* values) and associated effect sizes are reported in Supplementary Table S1.

**Table 2:** Histological analysis of residual debris. Residual debris area (%) per group, expressed as mean ± SD. Pairwise comparisons versus matched conventional controls report mean difference (Δ) with 95% confidence intervals (CIs) and standardised effect sizes (Hedges’ g with 95% CI)

Group	Residual Debris Area (%) ± SD	Δ vs. conventional (95% CI)	Hedges’ g (95% CI)
B1	45.8 ± 6.2	—	—
B2	12.4 ± 3.1*	−33.40 [− 38.13, − 28.67]	−6.53 [− 8.82, − 4.24]
B3	47.9 ± 5.8	—	—
B4	10.8 ± 2.9*	−37.10 [− 41.53, − 32.67]	−7.75 [− 10.42, − 5.08]

Statistics. Group differences were tested with the Kruskal–Wallis test (α = 0.05). Pairwise post hoc comparisons were performed using Dunn’s procedure (p adjusted). Omnibus non-parametric effect size: epsilon-squared (ε²) with 95% CI. Pairwise effect sizes: Hedges’ g (small-sample corrected). CIs for mean differences obtained using Welch’s method; CIs for Hedges’ g computed via the Hedges–Olkin normal approximation. Abbreviations: Δ, mean difference; CI, confidence interval. *p < 0.05 vs. conventional groups

**Table 3:** Confocal laser scanning microscopy (CLSM) analysis of NaOCl penetration depth. Penetration depth (µm) per group, expressed as mean ± SD. Pairwise comparisons versus matched conventional controls report mean difference (Δ) with 95% CIs and hedges’ g with 95% CI

Group	NaOCl Penetration Depth (µm) ± SD	Δ vs. conventional (95% CI), µm	Hedges’ g (95% CI)
C1	75 ± 15	—	—
C2	258 ± 22*	+ 183.00 [+ 165.15, + 200.85]	+ 9.31 [+ 6.15, + 12.46]
C3	70 ± 12	—	—
C4	275 ± 19*	+ 205.00 [+ 189.86, + 220.14]	+ 12.36 [+ 8.23, + 16.48]

Statistics. Kruskal–Wallis test (α = 0.05), with Dunn’s post hoc comparisons (p adjusted). Omnibus effect size: epsilon-squared (ε²) with 95% CI. Pairwise effect sizes: Hedges’ g (small-sample corrected). CIs as described in Table 2. Abbreviations: Δ, mean difference; CI, confidence interval. *p < 0.05 vs. conventional groups

## Discussion

The prespecified null hypothesis stated that no differences would exist among subgroups for any outcome within each analytical modality. The rejection of this hypothesis across all primary outcomes indicates that adjunctive activation with 3D Cleaning significantly modifies cleaning performance.

This ex vivo study compared the removal of smear layer and debris in traditional versus minimally invasive access cavities, using either conventional cleaning or the 3D Cleaning protocol. The success of endodontic therapy depends on multiple factors, including access cavity design, mechanical shaping, chemical cleaning, and obturation [14, 15]. Central to this process is the elimination of microorganisms and necrotic tissue from the root canal system, as well as the prevention of recontamination [16–18].

Chemical disinfection is indispensable because mechanical preparation alone cannot eradicate microorganisms. Nickel–titanium files generate a smear layer on canal walls [19,20,], which hinders the penetration of irrigants and the obturation process. Sodium hypochlorite (NaOCl) remains the irrigant of choice because of its antimicrobial activity, tissue-dissolving capacity, and cost-effectiveness [2,20,21], although it does not effectively remove the smear layer. For this reason, EDTA is widely used as a chelating agent [22].

Several methods have been proposed to activate irrigants. Ultrasonics remain the most popular, enhancing irrigant dynamics through acoustic streaming and cavitation [23]. Lasers have also been explored, but their cost and risk of irrigant extrusion beyond the apex are drawbacks [24]. Heating NaOCl to approximately 80 °C has been shown to increase its antimicrobial and tissue-dissolving effects [3, 11]. Iandolo et al. [25] reported that intracanal heating alone can effectively remove the smear layer and debris, even without the use of EDTA. The present study combined ultrasonic activation with intracanal heating of NaOCl within the pulp chamber, which yielded superior results compared with conventional protocols.

The importance of targeted pulp chamber decontamination has received little attention, even though debridement should logically begin in the chamber before extending to the canals. At the same time, minimally invasive endodontics has promoted conservative access designs to preserve peri-cervical dentine and improve resistance to fracture [26–28]. One of the main challenges of such designs is the potential retention of smear layer and residual tissue due to restricted irrigant dynamics [29]. Neelakantan et al. [30] found that conservative cavities were associated with more residual pulp tissue in pulp chambers compared with traditional designs, a finding consistent with the present study. In groups without activation (A1, A3), pulp chamber walls remained heavily coated with debris, whereas activation markedly reduced these deposits.

The main finding of this investigation was that ultrasonic activation of heated NaOCl significantly improved smear layer and debris removal, with the conservative access combined with 3D Cleaning achieving the most favourable outcomes. Histological analysis corroborated these results, showing lower residual debris in the activated groups, while CLSM demonstrated deeper NaOCl penetration into dentinal tubules. This enhanced penetration is particularly relevant for disrupting bacterial biofilms in areas inaccessible to mechanical instruments.

Although SEM remains a valuable tool for evaluating surface cleanliness, it requires specimen sectioning and provides semi-quantitative data subject to interpretation [30]. Future studies may benefit from micro-CT, which allows non-destructive three-dimensional analysis of canal cleanliness [31].

The present findings support the use of 3D Cleaning as a straightforward and effective adjunct, particularly when conservative access designs are selected. Nonetheless, these results derive from ex vivo experimentation and require confirmation through well-designed clinical trials.

Justification and safety considerations regarding NaOCl heating are important when interpreting the present findings. The 3D Cleaning protocol employed a System-B heat carrier with a setpoint of 180 °C; however, the irrigant was only transiently warmed to approximately 80 °C within the pulp chamber [11, 32]. The heat carrier tip was positioned 1 mm above the chamber floor, without contact with dentine, and activated for 8 s. This step was followed immediately by 20 s of ultrasonic activation. This cycle was repeated three times with complete irrigant renewal. Because the chamber was fluid-filled and highly dissipative, bulk NaOCl temperatures never approached the device setpoint and cooled rapidly after each pulse.

This short-pulse, non-contact strategy aligns with established thermal safety principles, as root surface temperature elevations remain below thresholds associated with periodontal injury when heat delivery is brief and spatially confined. From a mechanical perspective, in vitro data indicate that NaOCl heated to clinically relevant temperatures (approximately 70 °C) does not adversely affect dentine compressive strength compared with room-temperature use [33]. Collectively, these considerations support the rationale that transient intracanal warming of NaOCl to around 80 °C, immediately coupled with ultrasonic activation and frequent irrigant replacement, enhances cleaning efficacy while maintaining biological and mechanical safety.

### Limitations

The use of EDTA as part of a standardised, clinically representative protocol ensured uniform smear layer chelation but may have reduced observable contrasts attributable solely to irrigant activation. Future investigations should adopt a factorial design (EDTA vs. no EDTA × access design × activation protocol) and incorporate micro-CT and microbiological endpoints to disentangle chelation–activation interactions.

This study was conducted ex vivo, which facilitated rigorous control of variables but does not reproduce physiological conditions such as pulpal and periapical perfusion, periodontal ligament compliance, or body temperature. These factors may influence irrigant hydrodynamics, heat dissipation, and debris transport, thereby limiting direct clinical extrapolation of the present findings. Accordingly, the results should be interpreted as mechanistic evidence that requires confirmation in vivo and in clinical settings.

Furthermore, beyond morphological and penetration outcomes, this study did not evaluate microbiological endpoints (e.g. biofilm models, CFU counts, qPCR load reduction, CLSM live/dead analysis) or mechanical outcomes (e.g. fracture resistance or dentine property testing). Although published work suggests that NaOCl heated to clinically relevant temperatures does not impair dentine compressive strength, this requires validation under the specific timing and sequencing of the present protocol. Future studies should therefore adopt factorial designs integrating (i) quantitative microbiology, (ii) micro-CT–based morphometric analysis, and (iii) mechanical testing to determine whether enhanced cleaning translates into improved decontamination without compromising structural integrity.

### Clinical implications

Within the constraints of an ex vivo model, 3D Cleaning appears to be a practical adjunct when a conservative access design is selected. The protocol relies on standard clinical equipment, adds minimal operative time, and enhances both debridement and irrigant penetration, thereby helping clinicians balance structural preservation with effective disinfection.

## Conclusions

The present study demonstrated that the 3D Cleaning protocol substantially improves smear layer and debris removal, reduces residual tissue, and significantly enhances NaOCl penetration into dentinal tubules, irrespective of access cavity design. These findings underscore the potential of advanced irrigation strategies to optimise endodontic debridement, particularly when minimally invasive access is used to preserve coronal tooth structure. Further investigations, including micro-CT analyses and microbiological assessments, are warranted to validate and extend these results, thereby strengthening the clinical applicability of this protocol.

## Supplementary Information

Below is the link to the electronic supplementary material.

Supplementary file 1 (DOCX 29.6 KB)

## Data Availability

No datasets were generated or analysed during the current study.
