# Supplementary material for: SEM, confocal Laser, and histological evaluation of traditional and conservative access cavities with and without 3D cleaning: an ex vivo study
Source: Clin Oral Investig. 2025 Oct 11;29(11):499. doi: 10.1007/s00784-025-06606-9 (PMC12515115; doi:10.1007/s00784-025-06606-9)
Supplement: Supplementary file 1 — (DOCX 29.6 KB) [file 784_2025_6606_MOESM1_ESM.docx]

## Supplementary Table S1. Pairwise effect sizes and confidence intervals (CI) for histology and CLSM

Pairwise standardised effect sizes (Hedges’ g) and mean differences (Δ) with 95% CIs.

| Comparison | Outcome | Mean Difference (95% CI) | Hedges’ g (95% CI) |
| --- | --- | --- | --- |
| B2 vs. B1 | Residual debris (%) | −33.40 [−38.13, −28.67] | −6.53 [−8.82, −4.24] |
| B4 vs. B3 | Residual debris (%) | −37.10 [−41.53, −32.67] | −7.75 [−10.42, −5.08] |
| B4 vs. B2 | Residual debris (%) | −1.60 [−4.42, +1.22] | −0.51 [−1.37, +0.35] |
| B3 vs. B1 | Residual debris (%) | +2.10 [−3.54, +7.74] | +0.34 [−0.51, +1.18] |
| C2 vs. C1 | Penetration depth (µm) | +183.00 [+165.15, +200.85] | +9.31 [+6.15, +12.46] |
| C4 vs. C3 | Penetration depth (µm) | +205.00 [+189.86, +220.14] | +12.36 [+8.23, +16.48] |
| C4 vs. C2 | Penetration depth (µm) | +17.00 [−2.31, +36.31] | +0.79 [−0.09, +1.67] |
| C3 vs. C1 | Penetration depth (µm) | −5.00 [−17.82, +7.82] | −0.35 [−1.20, +0.49] |

Statistics. Group differences tested with Kruskal–Wallis (primary analysis, α = 0.05); pairwise post hoc with Dunn (p adjusted). Effect sizes: Omnibus non-parametric effect size reported as epsilon-squared (ε²) for Kruskal–Wallis; pairwise standardised effect sizes as Hedges’ g (small-sample corrected). Confidence intervals (CIs) for mean differences are obtained using Welch’s method, and CIs for Hedges’ g are computed via the Hedges–Olkin normal approximation. Abbreviations: Δ, mean difference; CI, confidence interval.
